# Improved *cis*-Abienol production through increasing precursor supply in *Escherichia coli*

**DOI:** 10.1038/s41598-020-73934-z

**Published:** 2020-10-08

**Authors:** Tao Cheng, Guang Zhao, Mo Xian, Congxia Xie

**Affiliations:** 1 A State Key Laboratory Base of Eco-Chemical Engineering, College of Chemistry and Molecular Engineering, Qingdao University of Science and Technology, Qingdao, 266042 China; 2 CAS Key Laboratory of Bio-Based Materials, Qingdao Institute of Bioenergy and Bioprocess Technology, Chinese Academy of Sciences, Qingdao, China

**Keywords:** Biochemistry, Biological techniques, Biotechnology, Microbiology

## Abstract

*cis*-Abienol, a natural diterpene-diol isolated from balsam fir (*Abies balsamea*), can be employed as precursors for the semi-synthesis of amber compounds, which are sustainable replacement for ambergris and widely used in the fragmented industry. This study combinatorially co-expressed geranyl diphosphate synthase, geranylgeranyl diphosphate synthase, Labda-13-en-8-ol diphosphate synthase and diterpene synthase, with the best combination achieving ~ 0.3 mg/L of *cis*-abienol. An additional enhancement of *cis*-abienol production (up to 8.6 mg/L) was achieved by introducing an exogenous mevalonate pathway which was divided into the upper pathway containing acetyl-CoA acetyltransferase/HMG-CoA reductase and HMG-CoA synthase and the lower pathway containing mevalonate kinase, phosphomevalonate kinase, pyrophosphate mevalonate decarboxylase and isopentenyl pyrophosphate isomerase. The genetically modified strain carrying chromosomal copy of low genes of the mevalonate with the trc promoter accumulated *cis*-abienol up to 9.2 mg/L in shake flask. Finally, *cis*-abienol titers of ~ 220 mg/L could be achieved directly from glucose using this de novo *cis*-abienol-producing *E. coli* in high-cell-density fermentation. This study demonstrates a microbial process to apply the *E. coli* cell factory in the biosynthesis of *cis*-abienol.

## Introduction

*cis*-Abienol, a bicyclic tertiary labdanoid diterpene alcohol found in balsam fir (*Abies balsamea*) and tobacco, is the fragrance precursor of most oriental tobaccos and part cigars, commonly used in cigarette extract^1–3^. It also plays a key role in the chemical defense against herbivores and pathogens, such as bark beetles and their associated fungi^4^. Furthermore, *cis*-abienol and other oxygen-containing diterpenoids of plant origin (e.g., sclareol and manool) can be used as precursors for the semi-synthesis of amber compounds like Ambrox^5^, which is a sustainable replacement for ambergris and widely used in the fragmented industry because of its high fixative and olfactory qualities^6,7^. Currently, the *cis*-abienol was extracted from *cis*-abienol plants^8,9^. The primary sources of *cis*-abienol are balsam fir^10^, tobacco trichomes (*Nicotiana tabacum*; family *Solanaceae*)^9,11^, or tuberous roots of Bolivian sunroot (*Polymnia sonchifolia*; family *Asteraceae*)^12^. The current isolation method of *cis*-abienol from the plant is inefficient, requiring substantial expenditure of natural resources and many of environmentally hazardous chemicals. So the problems of the shortage of natural resources and environmental pollution have let us employ microorganisms for the production of *cis*-abienol, which can utilize renewable glucose derived from lignocellulose.

**Figure 1:**
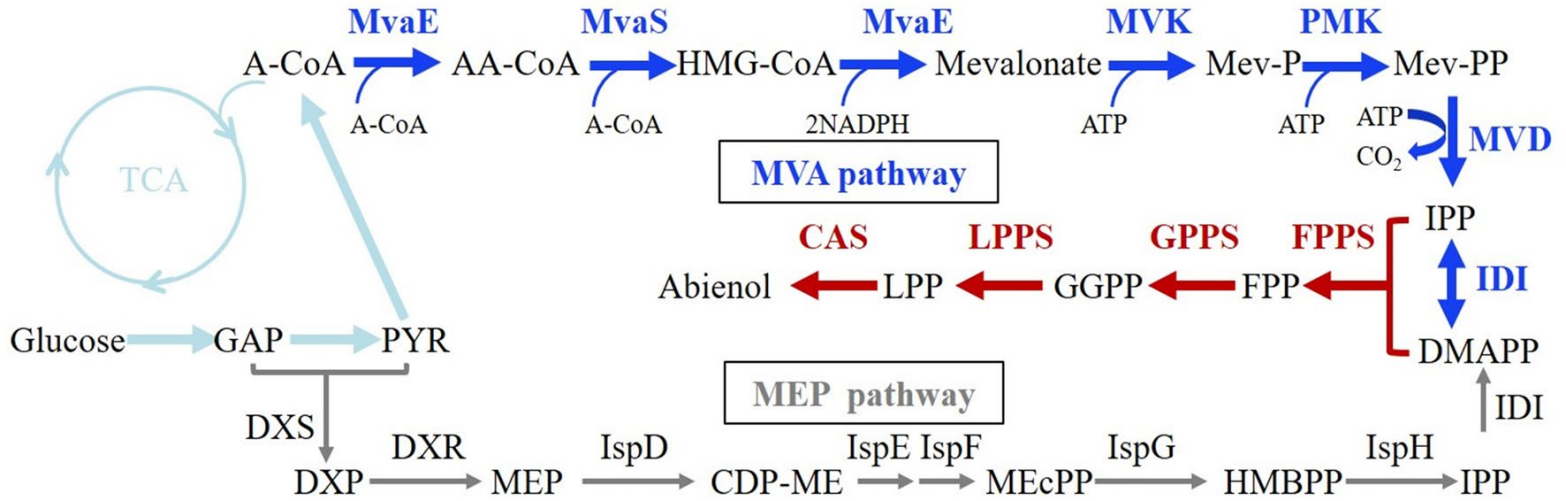
The MEP pathway and MVA pathway for *cis*-abienol production in *E. coli*. Genes overexpression are indicated in bold. Construction of the *cis*-abienol production strains overexpression the MVA pathway and *cis*-abienol pathway. Genes of MVA pathway are denoted by the blue arrows while the *cis*-abienol pathway is denoted by the violet red arrows. Enzymes involved in the MVA pathway and MEP pathway: MvaE, acetyl-CoA acetyltransferase/HMG-CoA reductase; MvaS, HMG-CoA synthase; MK, mevalonate kinase; PMK, phosphomevalonate kinase; MVD, mevalonate pyrophosphate decarboxylase; IDI, IPP isomerase; FPPS, farnesyl pyrophosphate synthase; GPPS, geranylgeranyl pyrophosphate synthase; LPPS, Labda-13-en-8-ol diphosphate synthase, CAS, *cis*-abienol synthase, Dxs, 1-deoxy-d-xylulose-5-phosphate synthase; Dxr, 1-deoxy-d-xylulose 5-phosphate reductoisomerase; IspD, 4-pyrophosphocytidyl-2-C-methyl-d-erythritol synthase; IspE, 4-pyrophosphocytidyl-2-C-methylerythritol kinase; IspF, 2-C-methyl-d-erythritol 2,4-cyclopyrophosphate synthase; IspG, 4-hydroxy-3-methylbut-2-enyl pyrophosphate synthase; IspH, 1-hydroxy-2-methyl-butenyl 4-pyrophosphate reductase. Pathway intermediates: GAP, glyceraldehyde 3-phosphate; PYR, pyruvate; DXP, 1-deoxy-d-xylulose 5-phosphate; MEP, 2C-methyl-d-erythritol 4-phosphate; CDP-ME, 4-diphosphocytidyl-2C-methylD-erythritol; MEcPP, 2C-methyl-d-erythritol 2,4-cyclodiphosphate; HMBPP, 1-hydroxy-2-methyl-2-(E)-butenyl 4-diphosphate; A-CoA, acetyl-CoA; AA-CoA, acetoacetyl-CoA; HMG-CoA, 3-hydroxy-3-methylglutaryl-CoA; Mev-P, mevalonate 5-phosphate; Mev-PP, mevalonate 5-diphosphate; IPP, isopentenyl pyrophosphate; DMAPP, dimethylallyl pyrophosphate; FPP, Farnesyl pyrophosphate; GGPP, geranylgeranyl pyrophosphate; LPP, Labda-13-en-8-ol diphosphate.

Microorganisms provide a sustainable and environment-friendly alternative for the production of *cis*-abienol starting from pure carbon sources and frequently show high product specificity^13^. *cis*-Abienol is synthesized from the cyclization of the geranylgeranyl diphosphate (GGPP) which is derived from isopentenyl pyrophosphate (IPP) and dimethylallyl pyrophosphate (DMAPP) manufactured through either the methylerythritol 4-phosphate (MEP) pathway in prokaryotes or the mevalonate (MVA) pathway in archaea/eukaryotes^14–16^. IPP is transformed into its isomer DMAPP by isopentenyl pyrophosphate isomerase (IDI). DMAPP and IPP are converted to farnesyl pyrophosphate (FPP) by farnesyl pyrophosphate synthase (ispA/Erg20) and further converted to GGPP by geranylgeranyl pyrophosphate synthase (CrtE) (Fig. 1). Biosynthesis of *cis*-abienol from GGPP involves two steps. The first is initiated by a carbon–carbon protonation, catalyzed by a class II diterpene synthase (diTPS) and leads to a cyclic diterpene diphosphate intermediate called labda-13-en-ol diphosphate (LDPP). The class II diTPS from *S. clare* has been cloned and functional characterized in *Escherichia. coli* for the biosynthesis of sclareol^17^. The second step of the pathway is catalyzed by *cis*-abienol synthase (Cas) which pertains to class I diTPS containing several types to produce a specific end product^18–20^. Based on this, the *cis*-abienol was biosynthesized in *Yarrowia lipolytica* by overexpressing the class II diPTS and Cas from different species^21^. Many biotechnological studies have been focused on the efficient production of desirable isoprenoids using *E. coli* as a host^22^. In *E. coli*, the isoprenoid was naturally biosynthesized through the endogenous MEP pathway^23^. For isoprenoid biosynthesis, a sufficient supply of IPP and DMAPP is essential. This has been often achieved by the expression of the idi and dxs (1-deoxy-d-xylulose 5-phosphate reductoisomerase) gene in the MEP pathway^24^. However, it has been shown that the introduction of the MVA pathway is more successful than engineering endogenous MEP pathway in *E. coli*, as it may avoid the natural regulatory mechanisms associated with the MEP pathway^25,26^. Michel et al. overexpressed the MVA pathway from *saccharomyces cerevisiae* and the enzymes responsible for the biosynthesis of sclareol to construct the sclareol biosynthetic pathway in genetically engineered *E. coli* and reached sclareol titers of ~ 1.5 g/L in high-cell-density fermentation^17^. Therefore, increasing the efficient supply of precursors is important to enhance *cis*-abienol production.

In this study, based on our previous work^27,28^, *cis*-abienol was produced by assembling a biosynthetic pathway in an engineering *E. coli*, using the heterologous MVA pathway combined with the additional of CrtE gene, Labda-13-en-8-ol diphosphate synthase (LPPS) gene from *Salvia. sclarea* and Cas gene from *A. balsamea*. The *cis*-abienol production of the final genetically modified strain, CM2/pCLES/pLCEC, was finally investigated under fed-batch fermentation condition. This study has begun the necessary foundations for a more sustainable route of *cis*-abienol production.

## Results

### Engineering a pathway for *cis*-Abienol biosynthesis

**Figure 2:**
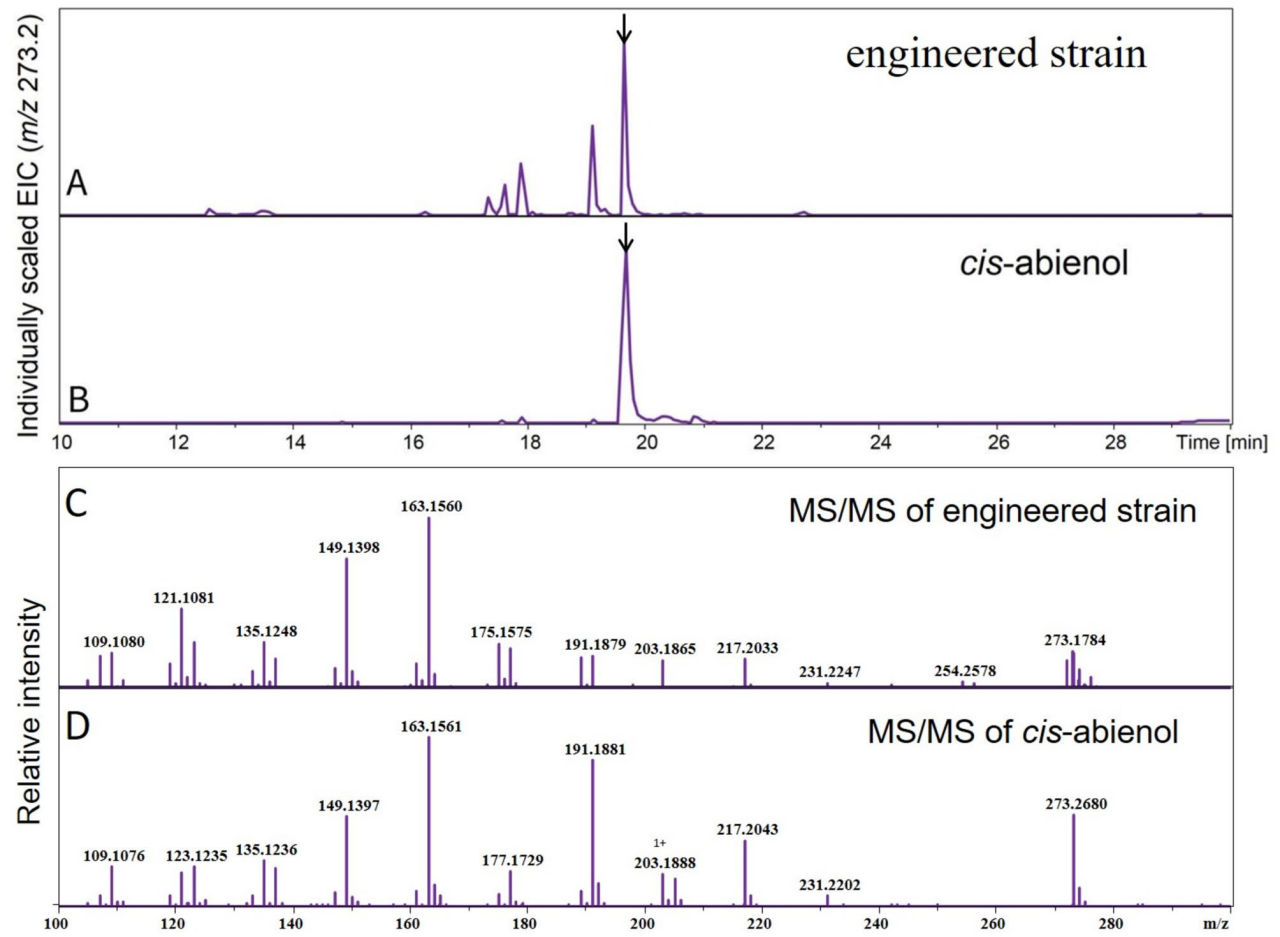
Identification of *cis*-abienol by LC–MS. (**A**) total ion current chromatogram of the extracts of the fermentation broth from *E. coli* BL21(DE3) harboring pLCCI after being induced for 48 h. (**B**) total ion current chromatogram of *cis*-abienol standard (the peak of *cis*-abienol was marked with an arrow corresponding to the retention time of 19.8 min). (**C**) mass spectrum of fermentation production. (**D**) mass spectrum of *cis*-abienol standard.

**Figure 3:**
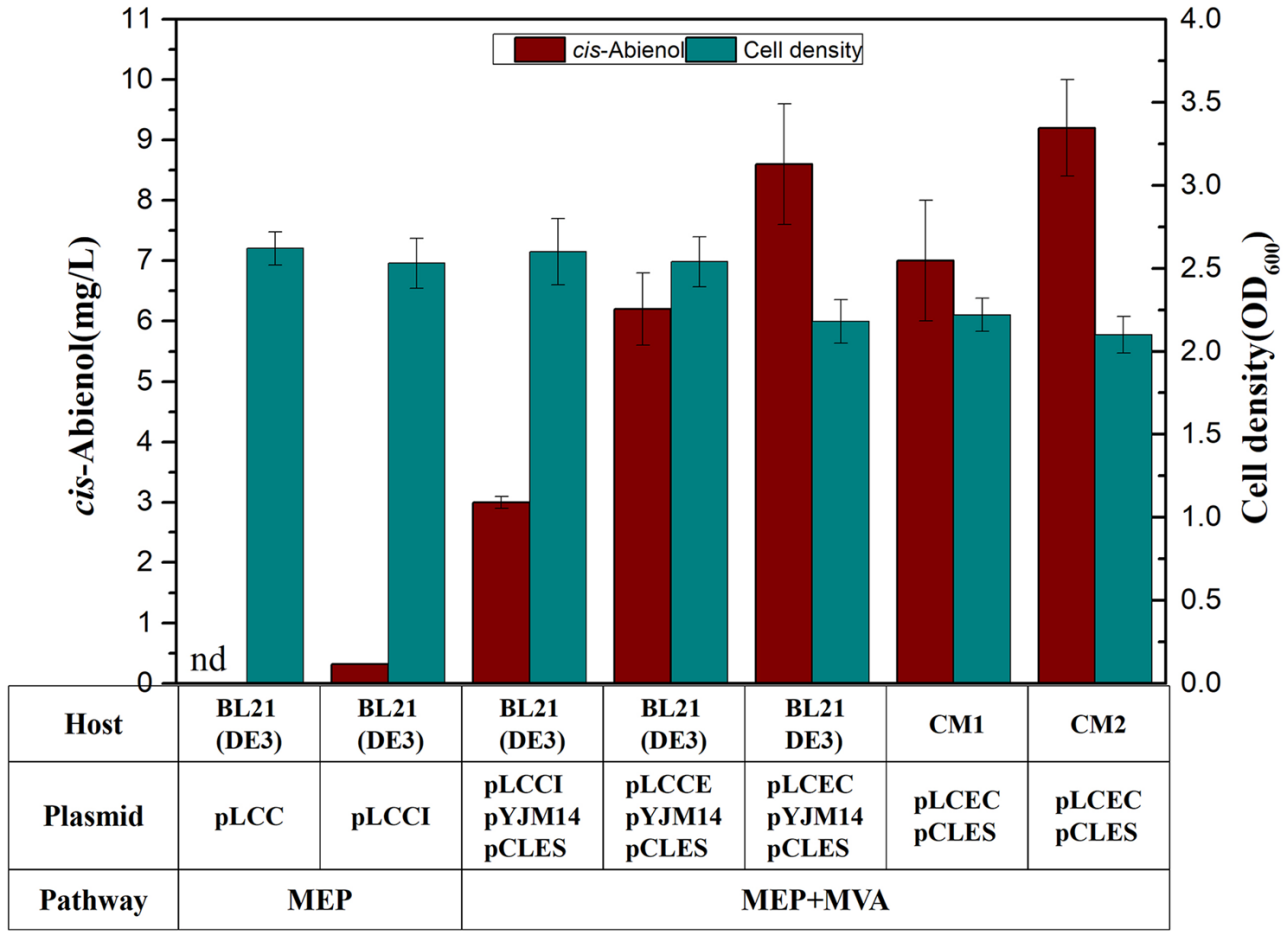
*cis*-Abienol production by BL21(DE3) harboring respective plasmid. CM1*, ERG8*, *ERG12*, *ERG19* and *IDI1*genes were integrated into the E. coli chromosome with trc promoter, CM2, *ERG8*, *ERG12*, *ERG19* and *IDI1*genes were integrated into the E. coli chromosome with Gi1.2 promoter, pLCC, pACYDuet-1 derivative carrying genes *Lpps, Cas, CrtE,* pLCCI, pACYDuet-1 derivative carrying genes *Lpps,Cas, CrtE, ispA*, pCLES*,* pCL1920 derivative carrying genes *mvaE*, *mvaS,* PYJM14, pTrcHis2B derivative carrying genes *ERG8*, *ERG12*, *ERG19* and *IDI1,* nd not detected. Data were obtained after each strain was induced for 48 h in liquid M9 mineral medium supplemented with 1 mM MgSO_4_ and 20 g/L glucose. Error bars represent the range of three independent fermentations.

GGPP, generated from either MEP or MVA pathway, could be catalyzed by the Lpps and Cas into *cis*-abienol. In the previous study^28^, it has proved feasible that overexpressing the *Lpps* gene and sclareol synthase gene along with the native MEP pathway to produce sclareol in *E. coli*. In this study, the *cis*-abienol biosynthesis pathway was constructed by expressing the primary enzymes diphosphate synthase (IspA), CrtE, Lpps, and Cas. The biosynthesis genes (*Lpps*, *Cas*, *CrtE*, *IspA*) of *cis*-abienol pathway were cloned into the plasmid pACYDuet-1 one by one to generate combination plasmid pLCCI. Accumulation of *cis-ab*ienol was not detected in the recombinant strain BL21 (DE3)/ pLCC for two days shake flask. We postulated that the low level of FPP may restrict the *cis-ab*ienol biosynthesis in the strain BL21(DE3)/pLCC. Because FPP pool was required to synthesize trans-octaprenyl diphosphate (ODP) and *cis*-undecaprenyl trans-undecapreyl diphosphate (UDP) in *E. coli*, which were the precursors of ubiquinone and peptidoglycan, respectively^29–31^. To improve the accumulation of FPP, the diphosphate synthase (IspA) gene was added to the plasmid to enhance the efficiency of the conversion of IPP and DMAPP to FPP. Thus BL21 (DE3) carrying pLCCI for two days shake flask resulted in one dominant peak in an LC–MS chromatogram (Fig. 2A) with a concentration of 0.32 mg/L of *cis*-abienol (Fig. 3). Comparison of the retention time and mass-spectrum of this compound with authentic standard confirmed the production of *cis*-abienol (Fig. 2A-D). The results showed that overexpressing the gene *LPPS, Cas, CrtE, IspA* together with the native MEP pathway was capable of synthesizing synthesize *cis*-abienol in *E. coli.*

### *cis*-Abienol microbial toxicity

**Figure 4:**
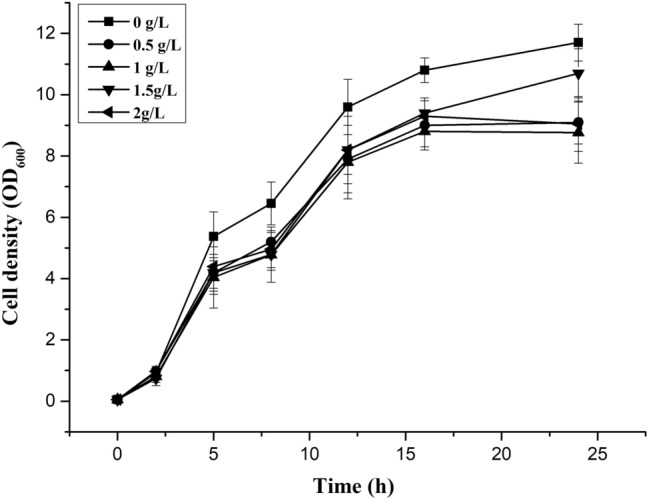
The growth of strain BL21(DE3)/pLCCI in LB medium with different concentrations of commercially available *cis*-abienol. The growth (OD_600_) was monitored for 36 h. *cis*-Abienol was added to the LB medium as follows: 0 g/L (■), 0.5 g/L (●), 1 g/L (▲), 1.5 g/L (▼) and 2 g/L (◄). Error bars represent the range of three independent experiments.

Oleoresin components, produced by conifers, most of which are a diverse of diterpenoids like *cis*-abienol, play a key role in the chemical defense against fungi^4^. Microbial production of *cis*-abienol in high titers may be restricted by its potential toxicity to the producing host. The effect of the concentration of *cis*-abienol on the *E. coli* strain was analyzed by measuring the OD_600_ for 36 h in shake flasks containing 20 mL of LB medium, which showed that *E. coli* strains could grow well at the commercially available *cis*-abienol titer from 0 g/L to 2 g/L. As shown in Fig. 4, when added exogenously to the growth medium, *cis*-abienol shows low toxicity to BL21(DE3)/pLCCI, the lack toxicity to the producing host should enable the production of *cis*-abienol in higher titers.

### Increasing the precursor supply for *cis*-Abienol biosynthesis

The low content of IPP and DMAPP also limits the efficient biosynthesis of terpenoids in *E. coli*. High-level polyprenyl pyrophosphate production may prove useful in producing a wide variety of compounds such as isoprene, pinene, longifolene, and sclareol^17,32–34^. One of the key strategies for constructing an efficient terpenoid biosynthesis host is to introduce a heterologous mevalonate pathway. Initially, our attempt to synthesize *cis*-abienol in *E. coli* by co-expressing genes *LPPS*, *Cas*, *CrtE* and *IspA* resulted in only a small amount of *cis*-abienol (~ 0.32 mg/L) in shake flask culture. To further improve production of *cis*-abienol, a metabolic engineering approach was adopted to increase the supply of precursor via overexpression of a heterologous mevalonate pathway into *E. coli*. The acetyl-CoA acetyltransferase/HMG-CoA reductase and HMG-CoA synthase from *Enterococcus faecalis* encoded by *mvaE* and *mvaS* were cloned into the low copy plasmid pCL1920 with medium-strength trc promoter to generate the recombinant plasmid pCLES. The plasmid pYJM14 containing the MVA lower pathway genes *ERG12*, *ERG8*, *ERG19* and *IDI1* from *S. cerevisiae* under the control of trc promoter (pTrcHis2B) has been constructed in the preliminary experiments in our laboratory^32^. The recombinant strain BL21 (DE3) (pCLES/pYJM14/pLCCI) carrying MVA pathway could accumulate 3.0 mg/L *cis*-abienol, which is about ten-fold of that (0.32 mg/L) produced by the control strain BL21 (DE3) (pLCCI) without MVA pathway (Fig. 3). According to the data obtained, increasing the supply of precursor of IPP and DMAPP by overexpression of MVA pathway is conducive to the *cis*-abienol biosynthesis.

In order to investigate the effect of FPP synthase *Erg20* of *S. cerevisiae* and different gene arrangement of *Erg20* and *CrtE* on the synthesis of *cis*-abienol, the recombinant pLCCE and pLCEC were constructed and transformed into BL21(DE3)/pCLES/pYJM14 competent cells, respectively. The strain carrying FPP synthase of *S. cerevisiae* produced 6.1 mg/L of *cis*-abienol, which is twofold to the strain with native FPP synthase. Although prior reports have shown that native FPP synthase is more effective than *S. cerevisiae* to produce sesquiterpene in *E. coli*^34^, we observed that the FPP synthase from *S. cerevisiae* was advantageous for the synthesis of *cis-ab*ienol. The reason may be that a high concentration of FPP was toxic to *E. coli* cell growth, resulting in a lower level of *cis-ab*ienol in BL21(DE3)/pCLES/pYJM14/pLCCI. This result demonstrated that the FPP synthase of *S. cerevisiae* was more efficient than the native one for FPP supply. We also found that different order for gene *Erg20* and *CrtE* leads to different production of *cis*-abienol. The strain BL21(DE3)/pCLES/pYJM14/pLCEC accumulated 8.6 mg/L of *cis*-abienol, and the yields were 1.5 times that of strain BL21(DE3)/pCLES/pYJM14/pLCCE (Fig. 3). It is concluded that the *FPP* and *CrtE* genes order in the operon affected relative expression levels and, consequently, the yield of *cis*-abienol production.

### Construction and characterization of chromosomal gene integration strain

Compared with the over-expression of plasmids, the synthesis of desired products by chromosomal integrated metabolic pathways has considerable advantages in industrial production^35^. The chromosomal integration of low pathway of MVA may improve the stability of exogenous genes in cells and reduce the use of antibiotics. The lower genes of MVA pathway *MVK, PMK, MVD, IDI* with Gi1.2 constitutive promoter^36,37^ and trc promoter were integrated into the *E. coli* chromosome to construct the strain CM1 carrying a chromosomal copy of Gi1.2-low genes and CM2 carrying a chromosomal copy of Trc-low genes, respectively. The integrated strain of CM1/pLCEC/pCLES resulted in a 30% decrease in *cis*-abienol production compared to the previous strain. In contrast, *cis*-abienol was accumulated up to 9.2 mg/L in the strain CM2/pLCEC/pCLES in shake flask (Fig. 3). It was 10% higher than the previous strain. The reason may be that CM1 with a synthetic promoter was too weaker to produce supplementary intermediate products IPP and DMAPP. Although a high copy plasmid (pYJM14) which contains the Erg8-ERg12-ERg19-IDI operon can provide a high copy number and stronger promoter, the replication of the plasmid and the expression of the genes may bring a heavy metabolic burden to the host. These indicate that only the suitable gene expression can increase the yield of the desired product. Due to less plasmid burden, the biomass of CM1 and CM2 was significantly higher than others strain.

### Bioreactor production of *cis*-Abienol

**Figure 5:**
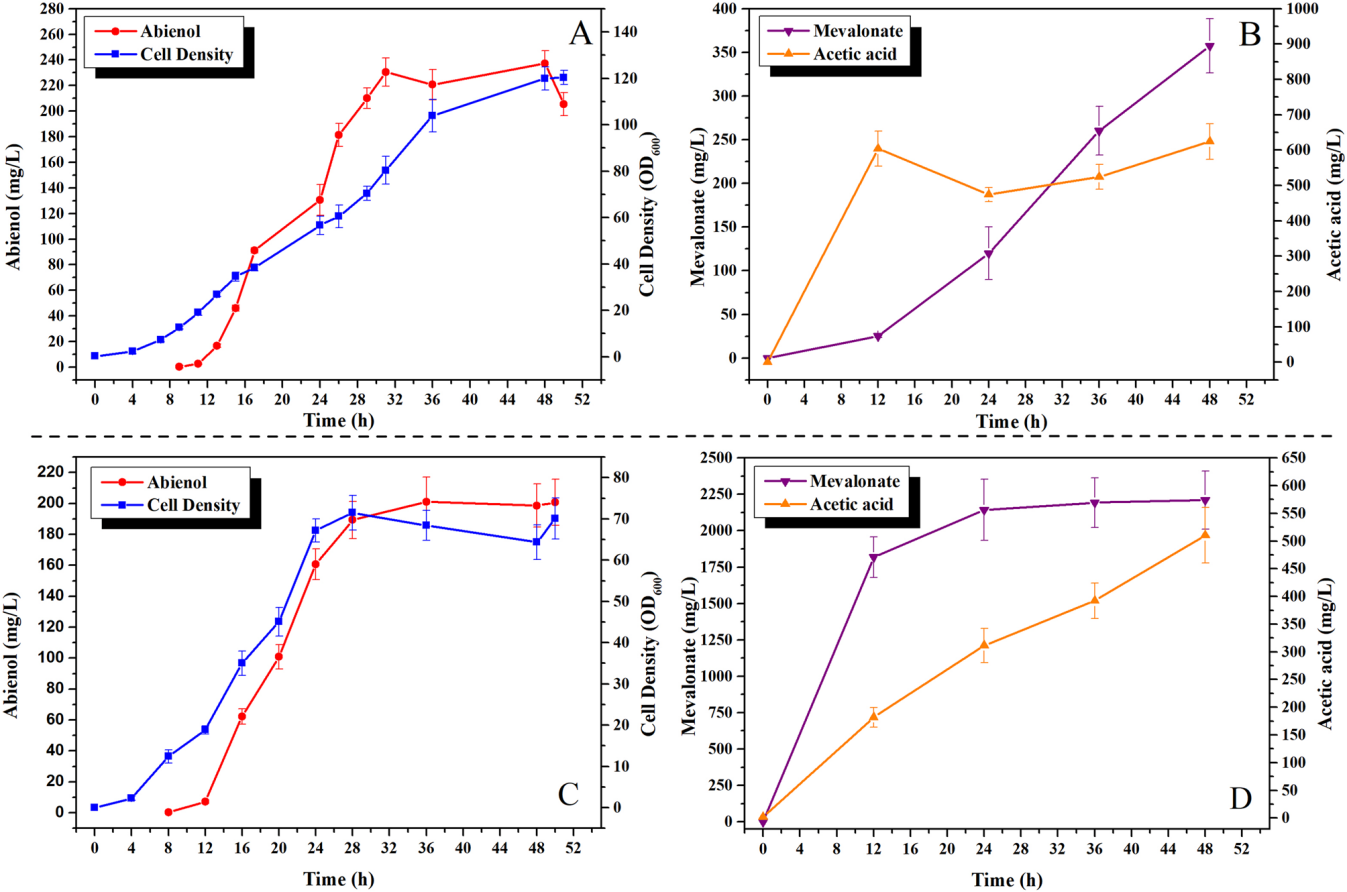
Time-course profiles for cell density (OD_600_), *cis*-abienol, mevalonate and acetic acid production during fed-batch fermentation of strain CM2/pLCEC/pCLES (**A**, **B**) and strain CM2/pLCEC/pETES (**C**, **D**). Cultures were performed in a 5 L stirred tank bioreactor. Error bars represent the range of three independent fermentations.

In order to investigate the performance of the most efficient strain CM2/pLCEC/pCLES, fed-batch cultivation experiments were performed in a 5-L laboratory-scale bioreactor containing 3L of M9 medium plus 0.5 g/L yeast extract. Glucose was fed at an appropriate rate to maintain it lower than 1 g/L. The biomass, *cis*-abienol production, and by-product (acetate and mevalonate) during the fermentation process were shown in Fig. 5A,B. After 50 h of fermentation, maximum cell densities reached an OD_600_ of 120, and *cis*-abienol titer reached a maximum of 220 ± 2 mg/L (Fig. 5A). The integration of the MVA downstream pathway into the genome may reduce the metabolic burden of the cells, obtaining higher biomass. Due to the low copy number (1 ~ 2) of plasmid (pCLES) in the upstream metabolic pathway of MVA in CM2/pCLES/pCLEC, the accumulation of intermediate metabolite mevalonate in the culture broth was only 357 mg/L (Fig. 5B). We postulated that lower mevalonate supply may affect the biosynthesis of *cis*-abienol.

To provide sufficient mevalonate, the CM2/pETES/pCLEC strain containing stronger promoter (T7) and higher copy numbers (~ 50) was constructed to reinforce the upper pathway of MVA. Under the same fermentation conditions as CM2/pCLES/pCLEC, maximum cell densities reached an OD_600_ of 70, *cis*-abienol titer reached a maximum of 200 mg/L (Fig. 5C) and the intermediate metabolite mevalonate accumulated in the culture broth and reached a maximum titer of 2200 mg/L (Fig. 5D). Due to the stronger promoter and higher copy numbers plasmid (pETES) in the upstream metabolic pathway of MVA in CM2/pCLES/pCLEC, the intermediate metabolite mevalonate increased in concentration by sixfold. However, cell biomass and the production of *cis*-abienol were significantly decreased. The excessive production of mevalonate in the upper pathway may exceed the catalytic capacity of the lower pathway, which leads to the accumulation of intermediate products. The metabolic unbalance between the upper and lower pathway resulted in miserable cell growth and decreased the production of *cis*-abienol. In our work, although CM2/pCLES/pCLEC strain enables the microbial biosynthesis of cis-abienol in high-cell-density fermentation, the *cis*-abienol production was relatively low. Future work should focus on optimizing expression levels of enzymes of Lpps and Cas to further increase *cis*-abienol production.

## Discussion

Diterpenes have attracted much attention owing to their unique structures and diverse bioactivities in recent years^38^. As a typical diterpene, the expanding applications and growing demands of *cis*-abienol, which was raw material for Ambrox synthesis and a spice additive in cigarettes, have led to a widespread search for effective and economical manufacturing methods. Microorganisms provide a sustainable and environment-friendly alternative for the production of terpenoids, starting from simple carbon sources and, frequently, ensuring high product specificity^13^. Many biotechnological studies have focused on using *E. coli* as host for efficiently producing desired isoprenoids, in particular, the introduction of heterologous MVA pathway genes into *E. coli* has been very effective in improving productivity. In this study, *E. coli* was used as a host to preliminarily verify the feasibility of producing *cis*-abienol by microorganisms.

The final reaction catalyzed by Cas should be the rate-limiting step in the biosynthesis of *cis*-abienol. Michel et al. overexpressed the enzymes responsible for the biosynthesis of sclareol and the MVA pathway from *S. cerevisiae* to construct a sclareol biosynthetic pathway in engineering *E. coli*, and achieved sclareol titers of about 1.5 g/L in high-cell-density fermentation. Due to the complex carbocation reaction to form bicyclic oxygen-containing diterpenoid, the activity of this enzyme is much lower than any other terpene synthase. However, in recent years, the advancements in comprehending the reaction mechanism of terpene synthase can guide the development of mutant *cis*-abienol enzymes with improved activity. Screening for effective *cis*-abienol synthase or using protein engineering strategies to improve its catalytic activity, which is currently being performed in our laboratories, have the potential to increase the productivity of this valuable diterpenes.

The low intracellular IPP and DMAPP content also restrict the efficient biosynthesis of terpenoids in *E. coli*. High-level yield of polyprenyl pyrophosphate has been demonstrated to be useful in the production of various compounds such as isoprene, pinene, longifolene, sclareol^17,32,33^. One of the Key strategies for constructing an efficient terpenoids biosynthesis host is introducing a heterologous mevalonate pathway. In this work, A hybrid MVA pathway was expressed in *E. coli* to promote the accumulation of IPP and DMAPP pools, thereby enhancing *cis*-abienol production to some extent. In order to improve the stability of foreign genes and reduce the use of the antibiotics, the lower genes of MVA pathway were integrated into the *E. coli* chromosome, and the production of *cis*-abienol has been further improved. It shows that further research on balancing the enzymes in MVA pathway will be beneficial for further increasing the production of *cis*-abienol. In the *cis*-abienol biosynthesis process, IPP and DMAPP are first condensed to form FPP, by FPP synthase, which is a critical enzyme in the mevalonate pathway, and then FPP condenses with IPP to form GGPP. After comparing two different FPP synthase, we established a complete pathway for the precursor biosynthesis, which further improved the yield of *cis*-abienol.

In this study, the best-performing strain CM2/pLCEC/pCLES was assessed in a shake flask with the M9 minimal medium and a 3L fed-batch fermentation with M9 fermentation medium. Cell density reached an optical density 2.3 and the *cis*-abienol concentration reached 9.2 mg/L (Fig. 3) in shake flask with M9 minimal medium, which was lower than 10.1 (cell density) and 41.1 mg/L (*cis*-abienol) with TB medium^39^. While the titer of *cis*-abienol in this study is lower than the previous report, the cell density in this study is much lower than in the last report^39^. Therefore, increasing cell density through optimization might lead to higher titers of *cis*-abienol. The maximum production of *cis*-abienol reached only 220 mg/L under fed-batch fermentation in the growth medium which was lower than 634.7 mg/L reported with two-phase cultivation in a fed-batch bioreactor. It may be that the higher content of *cis*-abienol in the broth hinders the spread out of the cell, thus exerting a negative feedback inhibition to the biosynthesis of *cis*-abienol. Therefore, the method of two-phase cultivation will be employed to improve the production of *cis*-abienol in future work.

In summary, we established a complete pathway to enhance the precursor supply of *cis*-abienol from simple building blocks. *cis*-Abienol production was achieved by assembling biosynthetic genes encoding a heterologous MVA pathway, an FPP synthase from *S. cerevisiae*, a GGPP synthase from *P. agglomerans*, Lpps and Cas from *A. balsamea* in an engineered *E. coli* strain. The yield of *cis*-abienol was further improved by fed-batch fermentation of the engineered strain. The best-performing strain CM2/pCLES/PLCEC accumulated *cis*-abienol up to 220 mg/L under fed-batch fermentation conditions. Although the production of *cis*-abienol in this study was not as high as previously reported, our study still shows a good strategy to use *E. coli* cell factory as a robust and competitive platform for the synthesis of *cis*-abienol.

## Methods

### Strains, media and culture conditions

Strains and plasmids used in this study were listed in Table 1. *S. cerevisiae* was used for gene cloning. The *E. coli* DH5α strain was used for the plasmid construction. The *E. coli* χ7213 was used for the chromosomal integration as the donor strain^40^, and *E. coli* BL21(DE3) was used for production of *cis*-abienol. *E. coli* DH5α and *E. coli* BL21(DE3) were cultured in Luria–Bertani (LB) broth with appropriate antibiotics added to the broth when necessary (50 μg/mL for kanamycin, 34 μg/mL for chloramphenicol, 100 μg/mL for ampicillin and 100 μg/mL for spectinomycin). *S. cerevisiae* was cultured in YPD medium. M9 minimal medium (15.3 g/L NaH_2_PO_4_·12H_2_O, 3 g/L KH_2_PO_4_, 1 g/L NH_4_Cl, 0.5 g/L NaCl) was used for shake flask fermentation. Growth medium (9.8 g/L K^2^ HPO_4_·3H_2_O, 0.5 g/L yeast extract, 2 g/L MgSO_4_·7H_2_O, 2.1 g/L citric acid monohydrate and 0.3 g/L ferric ammonium citrate) supplemented with 10 g/L glucose, and 1 mL/L trace elements (3.7 g/L (NH_4_)_6_Mo_7_O_24_·4H_2_O, 2.9 g/L ZnSO_4_·7H_2_O, 24.7 g/L H_3_BO_3_, 2.5 g/L CuSO_4_·5H_2_O, 15.8 g/L MnCl_2_·4H_2_O) was used for fed-batch fermentation as previously described^41^.

**Table 1:** Bacterial strains and plasmids used in this study.

Strain/plasmid	Relevant genotype/property	Source/reference
**Plasmids**		
pYJM14	pTrcHis2B derivative carrying genes *ERG8*, *ERG12*, *ERG19* and *IDI1*, Trc promoter, Ap^R^	^27^
pAC-lyc	pACYDuet-1 derivative carrying genes CrtE, CrtI, CrtB	^42^
pRE112	*ori*T *ori*V *sac*B cat	^40^
pRE112-ΔSU	pRE112 derivative carrying genes *glmS, glmU*	This study
pRE112-ΔSU-trc-low	pRE112 derivative carrying genes *glmS, glmU, ERG8*, *ERG12*, *ERG19* and *IDI1*, Trc promoter	This study
pRE112-ΔSU-Gi1.2-low	pRE112 derivative carrying genes *glmS, glmU, ERG8*, *ERG12*, *ERG19* and *IDI1*, Gi1.2 promoter	This study
pLCC	pACYDuet-1 derivative carrying genes *Lpps,Cas*, *CrtE*, T7 promoter, Cm^R^	This study
pLCCI	pACYDuet-1 derivative carrying genes *Lpps,Cas*, *CrtE, IspsA,* T7 promoter, Cm^R^	This study
pLCCE	pACYDuet-1 derivative carrying genes *Lpps,Cas*, *CrtE, Erg20,* T7 promoter, Cm^R^	This study
pLCEC	pACYDuet-1 derivative carrying genes *Lpps,Cas*, *Erg20, CrtE, *T7 promoter, Cm^R^	This study
pCLES	pCL1920 derivative carrying genes *mvaE*, *mvaS,* trc promoter, Spc^R^	This study
pETES	pET28a( +) derivative carrying genes *mvaE*, *mvaS*, T7 promoter, Kan^R^	This study
**Strains**		
*E. coli* BL21(DE3)	F^-^*ompThsdS*_*B*_(*r*_*B*_^*-*^*m*_*B*_^*-*^) *gal dcm rne131*(*DE3*)	Invitrogen
*E. coli Trans5α*	F-φ80 lac ZΔM15 Δ(lacZYA-arg F) U169 endA1 recA1 hsdR17(rk-,mk +) supE44λ- thi -1 gyrA96 relA1 phoA	Transgen Biotec
*E. coli* χ7213	Thi-1 thr-1 leuB6 fhuA21 lacY1 glnV44 *Δ*asdA4 recA1 RP4 2-Tc∷Mu[λpir] Km^r^	^40^
CM1	BL21(DE3)::Gi1.2-Low	This study
CM2	BL21(DE3)::Trc-Low	This study

### Plasmid and strain construction

All PCRs were done using PrimerSTAR Max DNA polymerase (TAKARA, Dalian, China). All of the primers used for DNA manipulation were listed in Table 2. The Labda-13-en-8-ol diphosphate synthase (*LPPS*, GenBank Accession No.: JQ478434.1) from *S. sclarea* and diterpene synthase TPS4 (*Cas*, GenBank Accession No.: JN254808.1) from *A. balsamea* were code optimized and synthesized by BGI. The *CrtE* gene were obtained from pAC-lyc ^42^. The geranyl diphosphate/farnesyl pyrophosphate synthase gene *ispA* and *Erg20* was obtained from *E. coli* and *S. cerevisiae*, respectively. Four genes of the enzymes LPPS, CAS, CrtE, ispA were amplified and cloned into the plasmid pACYDuet-1. The gene for LPPS was amplified by PCR with primers and cloned into *Bam*HI and *Sac*I sites of vector pACYDuet-1and the resultant plasmid was named pACY-Lpps. The gene for CAS was amplified by PCR with primers and cloned into *Bam*HI and *Sac*I sites of vector pACY-LPPS to generate recombinant plasmid pACY-LPPS-Cas. The gene *crtE* and *ispA* were amplified with primers and ligated using overlapping PCR to generate an engineering fragment, which was cloned to the plasmid pACY-LPPS-Cas to generate recombinant plasmid pLCCI. The *crtE* and *Erg20* gene were amplified with primers and ligated using overlapping PCR to generate an engineering fragment, which was cloned to the plasmid pACY-LPPS-Cas to generate recombinant plasmid pLCCE. To construct pLCEC, *Erg20* and *CrtE* were amplified with primers and ligated using overlapping PCR to generate an engineering fragment and cloned into the plasmid pLC at *Aat*II and *Xho*I. To construct pTES, mvaE-mvaS was amplified from pACY-mvaE-mvaS which was constructed in our previous work and cloned into pTrchis2B at *Sac*I and *Nco*I. To construct pLCES, trc-mvaE-mvaS was amplified from pTES and cloned into pCL1920 using an In-Fusion HD Cloning Kit (Takara-Clontech, Japan). To construct pETES, mvaE-mvaS was amplified from pACY-mvaE-mvaS and cloned into pET28 at *Sac*I and *Nco*I.

**Table 2:** Primers used in this study for plasmid and mutant strain construction.

Oligonucleotide primers	sequence
Lpps_F_*Bam*HI	CGCGGATCCGATGACCAGCGTGAATTTA
Lpps_R_*Sac*I	CGCGAGCTCTTACACCACCGGGCGAAAC
Cas_F_*Sac*I	CGCGAGCTCCTTAAGGTAGCTGCATGCA
Cas_R_*Hin*dIII	CCCAAGCTTTTAGGTCGCCGGTTCAAAC
CrtE_F_*Xho*I	CTCGAGATGGTGAGTGGCAGTAAAGC
CrtE_R	AGATCTGCCATATGTATATCTCCTTTCAGGCGATTTTCATGACCG
IspA_F1	AAGGAGATATACATATGGCAGATCTCATGGACTTTCCGCAGCAACT
IspA_R_*Pac*I	TTAATTAATTATTTATTACGCTGGATGATGTAG
Erg20_F_1	AAGGAGATATACATATGGCAGATCTCATGGCTTCAGAAAAAGAAAT
Erg20_R_*Pac*I	TTAATTAACTATTTGCTTCTCTTGTAAACTTT
Erg20_F_*Xho*I	GGACTCGAGCATGGCTTCAGAAAAAGAAATTAGGAGAGAGAG
Erg20_R	GCTCTCGCCAGGTTCTGAAGCAGTTCTATTTGCTTCTCTTGTAAAC
CrtE_F	AACTGCTTCAGAACCTGGCG
CrtE_R_*Pac*I	TTAATTAATCAGGCGATTTTCATGACCG
ES_F	GATTCATTAATGCAGCTGCTGAAATGAGCTGTTGACAA
ES_R	TCGCTATTACGCCAGCTGATTGAAGCATTTATCAGGGTT
pCL_F	CAGCTGGCGTAATAGCGA
pCL_R	CAGCTGCATTAATGAATC
ES_F_*Nco*I	CCATGGAGATGAAAACAGTAGTTATTATTGATGC
ES_R_*Xho*I	GGCTCGAGTTAGTTTCGATAAGAGCGAACGG
glmS_F_*Xba*I	TGCTCTAGATGTCACAGTCTGGCGAAACCG
glmS_R	TTTGATTAAAACTCGAGCGGGCGGCCGCAAATAAGAAAAATGCCCCGCTTACG
glmU_F	GCATTTTTCTTATTTGCGGCCGCCCGCTCGAGTTTTAATCAAACATCCTGCCAACTC
gluU_R_*Kpn*I	CGGGGTACCTGTTTTTCCACTCTTCGTTCACTTT
Trc-low_F_*Not*I	GATCGCGGCCGCGCCCTTGACGATGCCACATCCTGAGCAAATAATT
Gi1.2_MVK_F_*Not*I	GATCGCGGCCGCGCCCTTGACGATGCCACATCCTGAGCAAATAATTCAACCACTAATTGTGAGCGGATAACACAAGGAGGAAACAGCTATGTCATTACCGTTCTTAACTTCTGCACCGGG
Ter_IDI_*Xho*I	GCCGCTCGAGCACGTCATCTGACGTGCCTTTTTTATTTGTAGACGCGTTGTTATAGCATTCTATGAATT

The restriction sites in the primers were underlined.

### Construction of BL21(DE3) strains with chromosomal mutation

The mutant strain was construction using suicide plasmid pRE112 as previously described^40^. To insert the lower pathway of MVA into *E. coli* BL21 (DE3) chromosome, a set of suicide plasmid was constructed based on the vector pRE112. For example, the flanking regions of *glmS* and *glmU* gene were amplified and linked up with each other by overlap extension PCR, and the restriction sites of *Not*I and *Xho*I were introduced at the connection point by primer design. This fragment was cloned into the vector pRE112 to generate pRE112-ΔSU. Then a *Not*I-*Kpn*I fragment from pYJM14, encoding lower pathway of MVA with Trc promoter or Gi1.2 constitutive promoter, was inserted into the corresponding site of pRE112-ΔSU, and the resulting plasmid was defined as pRE112-ΔSU-Trc-low or pRE112-ΔSU-Gi1.2-low, which was used to mediate the allelic exchange. After two rounds of selection, based on the positive marker chloramphenicol resistance gene *cat* and negative marker levan-sucrose gene *sacB* from *Bacillus *spp.^43^, we obtained the strain CM1 carrying chromosomal copy of low genes with gi1.2 promoter and the strain CM2 carrying chromosomal copy of low genes with Trc promoter.

### Shake flask cultivation

A single clone was inoculated into 3 mL of LB medium containing appropriate antibiotic and cultivated at 37 °C, 180 rpm for about 6 h. Then 1 mL of culture was used to inoculate into 500 mL saline bottle containing 100 mL M9 minimal medium with 2.0% (w/v) glucose as the main carbon source and cultivated at 37 °C, 180 rpm. 0.5 mM of IPTG was added to induce recombinant proteins expression at an OD_600_ reached about 0.6. Then the temperature was shifted to 30 °C for *cis*-abienol production. The cell mass and *cis*-abienol were determined after 48 h culturing.

### *cis*-Abienol microbial toxicity

To investigate the cytotoxicity of *cis*-abienol to the producing *E. coli* strain, LB medium was adjusted to the six different *cis*-abienol concentrations ranging from 0 to 2 g/L by adding various amounts of a highly concentrated *cis*-abienol micellar solution (50 g/L). Subsequently, the medium (10 mL in 100 mL flask) was inoculated with 5% of preculture grown overnight in LB and incubate at 37 °C under agitation (200 rpm) for 24 h. For each concentration of abieonl, the maximum specific growth rate was determined.

### Production of *cis*-Abienol by fed-batch

A single colony was picked and used to inoculate 3 mL LB medium supplemented with 34 μg/mL of chloramphenicol and 100 μg/mL of spectinomycin and cultivated at 37 °C, 180 rpm for about 6 h. Afterwards, 1 mL of the culture was used to inoculate a 100 mL M9 minimal medium containing appropriate antibiotics in a 500 mL shake flask and cultivated at 37 °C, 180 rpm for about 8 h. The seed culture was used in a ratio of 3% (v/v) to inoculate a 5-L fermenter containing 3L M9 fermentation medium. Sparger aeration was employed using filtered air to maintain the dissolved oxygen (DO) concentration The fermentation process was operated under the following conditions: temperature 32 °C, pH was controlled at 7.0 ± 0.1 by automatic addition of 25% ammonia water, agitation rate at 400 rpm and airflow at 1 vvm. The agitation was associated with DO to maintain a DO concentration above 30% saturation. When the initial glucose was depleted, 60% (v/v) concentrated glucose intermittently fed into the fermenter to maintain the residual glucose below 1 g/L to control the formation of acetic acid. The culture was induced when the OD_600_ was 12 by the addition of 0.25 mM IPTG unless specified according to different purposes. Samples were collected at certain intervals for cell density, residual glucose, acetic acid, mevalonate and *cis*-abienol analysis.

### Analytical methods

Cell densities of the cultures were determined by measuring optical density at 600 nm using a spectrophotometer (Cary 50 UV–vis, Varian). Cell density samples were diluted as necessary so as to fall within the linear range.

The residual glucose in the culture broth was determined using a Biochemistry Analyzer (YSI 2950D, YSI Life sciences, USA).

The concentration of acetic acid and mevalonate were determined in an Agilent 1200 Infinity series HPLC system (Agilent, Santa Clara, CA), coupled with an Aminex HPX-87H column (Bio-Rad, Hercules, CA) heated at 50 °C and a 1 cm precolumn. The mobile phase of 5 mM H_2_SO_4_ was run at 0.5 mL/min. A differential refractive index detector (Agilent, Santa Clara, CA) was used for analyte detection and quantification.

*cis*-Abienol was determined using an Ultimate 3000 UHPLC (Thermo, USA) quipped with an ultraviolet (UV, 237) detector and a Thermo Acclaim RSLC C18 column (2.1 mm × 100 mm, 2.2 μm) based on a method adapted from Minghong Gao et al.^44^ Measurement were performed at a column temperature of 30 °C and a flow rate of 0.2 mL/min. A solvent gradient of 0.1% of formic acid in water (A) and ACN (B) with 0.1% formic acid starting at 2% B to 100% B in 15 min and hold for 10 min, returning to 2% B at 25.1 min, holding these conditions at 30 min and stopping the controller. Mass spectrometry (Q-TOF) system (Bruker Daltonics, Billerica, USA) was performed in positive mode measuring ESI ionization, using the following operation parameters: capillary voltage 4500 V, dry temperature 200 °C, nebulizing gas of 1.5 bar, drying gas (N_2_, purity 99.999%) flowing of 4.5L/min. High resolution MS and MS/MS spectra were acquired in the range 50–1300 m*/z*. Otof Control software was used to carry out mass spectrometer control and data acquisition and Compass Data Analysis software was applied for data analysis.
